# Nitrogen and hydrophosphate affects glycolipids composition in microalgae

**DOI:** 10.1038/srep30145

**Published:** 2016-07-21

**Authors:** Xin Wang, Zhouyuan Shen, Xiaoling Miao

**Affiliations:** 1 State Key Laboratory of Microbial Metabolism and School of Life Sciences & Biotechnology, Shanghai Jiao Tong University, 800 Dongchuan Road, Shanghai 200240, China; 2 Biomass Energy Research Center, Shanghai Jiao Tong University, Shanghai 200240, China

## Abstract

Glycolipids had received increasing attention because of their uses in various industries like cosmetics, pharmaceuticals, food and machinery manufacture. Microalgae were competitive organisms to accumulate metabolic substance. However, using microalgae to produce glycolipid was rare at present. In this study, glycolipid content of *Chlorella pyrenoidosa* and *Synechococcus* sp. under different nitrate and hydrophosphate levels were investigated. The highest glycolipid contents of 24.61% for *C. pyrenoidosa* and 15.37% for *Synechococcus* sp. were obtained at nitrate absence, which were 17.19% for *C. pyrenoidosa* and 10.99% for *Synechococcus* sp. at 0.01 and 0 g L^−1^ hydrophosphate, respectively. Glycolipid productivities of two microalgae could reach at more than 10.59 mg L^−1^ d^−1^. Nitrate absence induced at least 8.5% increase in MGDG, DGDG and SQDG, while hydrophosphate absence resulted in over 21.2% increase in DGDG and over 48.4% increase in SQDG and more than 22.2% decrease in MGDG in two microalgae. Simultaneous nitrate and hydrophosphate limitation could make further improvement of glycolipid accumulation, which was more than 25% for *C. pyrenoidosa* and 21% for *Synechococcus* sp. These results suggest that nitrogen and phosphorus limitation or starvation should be an efficient way to improve microalgal glycolipid accumulation.

Glycolipid is a kind of a complex carbohydrate made of sugar and fat by covalently bound, it can be divided into two categories: sphingoglycolipid and glyceroglycolipid. Sphingoglycolipid mainly exists in animals, while glyceroglycolipid is widely found in higher plants, algae and bacteria. In thylakoid membranes, the majority (80–90%) of lipids are glycolipids^1^,^2^, including monogalactosyldiaclyglycerol (MGDG; 40–50% of thylakoid membrane lipids), digalactosyldiacylglycerol (DGDG; 20–30%) and sulfoquinovosyldiacylglycerol (SQDG; 20–30%)^1^. Glycolipid is a class of amphiphilic compounds, it plays an important role in creating well-ordered biological systems, and closely relate to the exchange of energies, substances and signals^3^. Due to the amphiphilic character, glycolipid being used as a kind of biosurfactant gained extensive attention^4^. Glycolipid can be also widely used in various industries like cosmetics, pharmaceuticals, food and machinery manufacture. At present, glycolipids with good application to food, medicine, etc., have been extracted from animals, plants and microorganisms. Microalgae are rich in glycolipid. And also, microalgae have the advantages of simple culturing, rapid growth, capacity of photosynthesis, etc. Thus, using microalgae to produce glycolipid received widely attention. Some glycolipids that have pharmacological activity have been isolated from microalgae cells. For example, a kind of sulfate-group containing glyceroglycolipid was separated from the cell extracts of cyanobacteria *L. lagerheimii* by Gustafson *et al*.^5^, which can inhibit the replication of HIV; two kinds of glyceroglycolipid MGDG that isolated from freshwater green algae *Chlorella vulgaris*, have a strong role in inhibiting tumor growth^6^; some of glyceroglycolipid isolated from the marine dinoflagellate have hemolytic activity^7^. Due to the structural diversity, many unknown glycolipids have not been studied.

The growth and metabolic substance accumulation of microalgae can be affected greatly by environmental factors. For example, light intensity, salinity, nitrogen concentration and iron content could affect microalgae lipid accumulation^8^,^9^,^10^; nitrogen starvation could result in an increase in triacylglycerol content^11^,^12^. Thus, altering the environmental factors of microalgae cell growth can be an effective way to obtain metabolic substance. Unlike the studies undertaken by researchers to explore the lipid accumulation and fatty acid composition in microalgae, studies on glycolipid accumulation of microalgae are seldom. Damiani *et al*.^13^ analyzed lipid in *Haematococcus pluvialis*, found that nitrogen deficiency could lead to accumulation in glycolipid content to about 6.67%. Liang *et al*.^14^ studied the effects of phosphorus on lipid accumulation in *Chlorella* sp., found that phosphorus limitation could increase glycolipid content to 10%. The content of three main kinds of glycolipid MGDG, DGDG and SQDG in microalgae could also be affected by nitrogen and phosphorus concentration^15^,^16^,^17^. The glycolipid contents in these studies were not that high, it still could be further improved. As glycolipid has great values in many aspects, it is very essential to analyze the glycolipid accumulation of microalgae in order to further industrial production.

According to our previous research^18^, *Chlorella pyrenoidosa* shows advantages like fast growth and high lipid content. *Synechococcus* sp. also has a high content of lipid based on our preliminary experiments. Glycolipid is a part of lipid, and it can be found in both of the microalgae. Therefore, they were chosen in the present research to evaluate the glycolipid accumulation of microalgae under different nitrate and hydrophosphate levels, with a focus on the changes of three glycolipid contents, and optimize a suitable condition for glycolipid accumulation.

## Results and Discussion

### Growth of *C. pyrenoidosa* and *Synechococcus* sp. subjected to different nitrate and hydrophosphate concentrations

**Figure 1:**
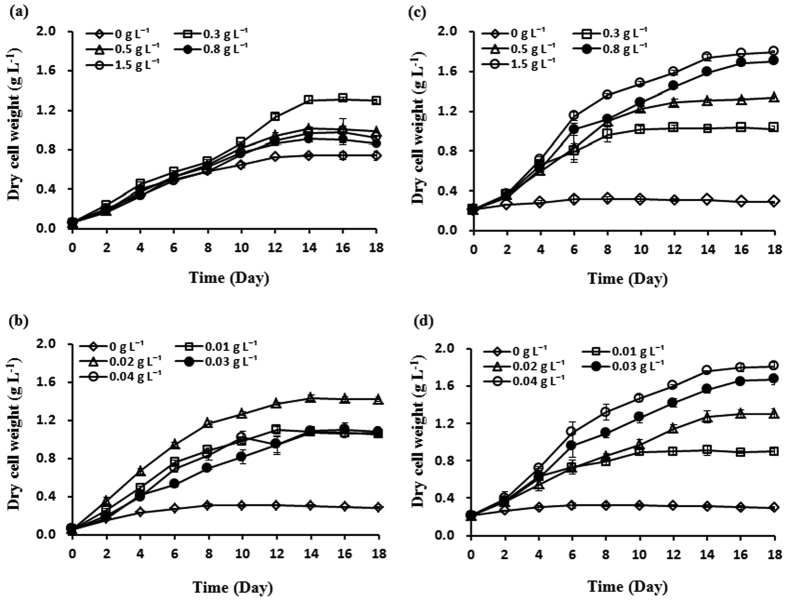
The growth of *Chlorella pyrenoidosa* and *Synechococcus* sp. cultivated in modified BG-11 medium at 25 ± 2 °C and 140 μmol/m^2^/s under different nitrate and hydrophosphate concentrations. (**a**) The growth of *Chlorella pyrenoidosa* under different nitrate concentrations. (**b**) The growth of *Chlorella pyrenoidosa* under different hydrophosphate concentrations. (**c**) The growth of *Synechococcus* sp. under different nitrate concentrations. (**d**) The growth of *Synechococcus* sp. under different hydrophosphate concentrations (means ± SD).

The effects of different nitrate and hydrophosphate concentrations on the growth of *C. pyrenoidosa* and *Synechococcus* sp. were shown in Fig. 1. The reduction of nitrate and hydrophosphate concentration in culture medium ranging from 1.5 to 0 g L^−1^ and 0.04 to 0 g L^−1^, respectively, induced a decrement in biomass production. The biomass concentrations of *C. pyrenoidosa* and *Synechococcus* sp. under 0 g L^−1^ nitrate concentration were only 0.74 g L^−1^ and 0.28 g L^−1^, respectively (Fig. 1a,c). Under 0 g L^−1^ hydrophosphate concentration, the biomass concentrations of *C. pyrenoidosa* and *Synechococcus* sp. decreased to 0.31 g L^−1^ and 0.29 g L^−1^, respectively (Fig. 1b,d). The similar phenomenon has also been found in some other microalgae species such as *Scenedesmus* sp. LX1^19^, *Chlorella* sp. F&M-M48^20^, etc. Nitrogen is one of the essential nutrients for microalgae cell growth, it is necessary for the synthesis of vivo protein (e.g., structural proteins, a variety of carriers and enzymes), nucleic acids and chlorophyll^21^. Phosphorus is the main component of nucleic acids, proteins and phospholipids, and it is also necessary for the synthesis of chlorophyll^22^. Microalgae must ingest nitrogen and phosphorus from the outside to meet the needs of growing.

It is interesting to note that biomass concentration either maintained or increased slightly under different nitrate concentrations (0, 0.5, 0.8 and 1.5 g L^−1^) for *C. pyrenoidosa* (Fig. 1a). However, *Synechococcus* sp. did not show the similar regulation in growth curve under different nitrate concentrations (Fig. 1b). As showed in Table 1, for *C. pyrenoidosa*, the maximum biomass concentration under different nitrate concentrations declined from 0.97 (1.5 g L^−1^) to 0.74 g L^−1^ (0 g L^−1^), the changes were little; but for *Synechococcus* sp., the maximum biomass concentration declined from 1.81 (1.5 g L^−1^) to 0.28 g L^−1^ (0 g L^−1^), the changes were significant. The maximum biomass concentration of *C. pyrenoidosa* (1.30 g L^−1^) and *Synechococcus* sp. (1.81 g L^−1^) were found in cells grown in nitrate concentration of 0.3 g L^−1^ and 1.5 g L^−1^, which were 1.8 and 6.5 times than that in 0 g L^−1^ nitrate concentration (0.74 and 0.28 g L^−1^), respectively (Table 1). Previous studies had also found in other Chlorella strains, such as *C. vulgaris*, *C. emersonii* and *C. sorokiniana*^23^, which low nitrogen concentration lead to higher biomass concentrations. Przytocka-Jusiak *et al*.^24^ also reported that rich nitrogen concentration caused an inhibition of cell division for *C. vulgaris*. These results suggested that *C. pyrenoidosa* be able to grow in nitrogen limitation or starvation conditions, while *Synechococcus* sp. be more stringent with nitrogen source then *C. pyrenoidosa*.

Under different hydrophosphate levels, biomass concentration was either maintained or increased slightly at 0.01, 0.03 and 0.04 g L^−1^ hydrophosphate for *C. pyrenoidosa* (Fig. 1c), which was different from *Synechococcus* sp. (Fig. 1d). As showed in Table 1, the maximum biomass concentration of *C. pyrenoidosa* (1.44 g L^−1^) and *Synechococcus* sp. (1.83 g L^−1^) were found in cells grown in hydrophosphate concentration of 0.02 g L^−1^ and 0.04 g L^−1^, which were 4.7 and 6.3 times than that in 0 g L^−1^ hydrophosphate concentration, respectively. It also could be found that the maximum biomass concentration for *C. pyrenoidosa* declined from 1.08 (0.04 g L^−1^) to 0.31 g L^−1^ (0 g L^−1^), the change was significantly compared to nitrogen rich (0.97 g L^−1^) and deficiency (0.74 g L^−1^) conditions (Table 1). These results suggested that *C. pyrenoidosa* also be able to grow in phosphorus limitation conditions and phosphorus starvation have greater influence on biomass concentration of *C. pyrenoidosa* than nitrogen starvation.

### Effect of different nitrate and hydrophosphate concentrations on glycolipid and lipid content

**Figure 2:**
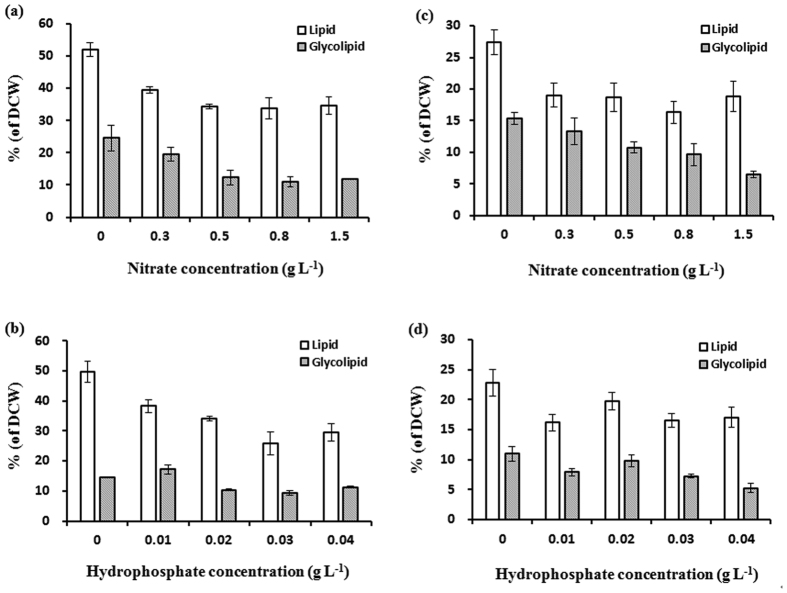
Effects of different nitrate and hydrophosphate concentrations on glycolipid and lipid contents. (**a**) Effects of different nitrate concentrations on glycolipid and lipid contents of *Chlorella pyrenoidosa.* (**b**) Effects of different hydrophosphate concentrations on glycolipid and lipid contents of *Chlorella pyrenoidosa.* (**c**) Effects of different nitrate concentrations on glycolipid and lipid contents of *Synechococcus* sp. (**d**) Effects of different hydrophosphate concentrations on glycolipid and lipid contents of *Synechococcus* sp. (means ± SD).

The major lipid species in these two algal strains are neutral lipids and polar lipids. Neutral lipids were mainly consisted of triacylglycerols (TAG) (11.38% of dry cell weight for *C. pyrenoidosa* and 4.72% for *Synechococcus* sp.). Polar lipids were mainly consisted of glycolipids and phospholipids (6.38% for *C. pyrenoidosa* and 7.65% for *Synechococcus* sp. of phospholipids). In this study, we mainly analyzed the glycolipid contents of the two microalgae. The glycolipid and lipid contents of *C. pyrenoidosa* and *Synechococcus* sp. under different nitrate and hydrophosphate concentrations were shown in Fig. 2. As shown in Fig. 2, nitrogen and phosphorus limitation or starvation is an effective way for glycolipid accumulation in both of these two microalgae. With the reduction of nitrate concentration ranging from 1.5 to 0 g L^−1^, glycolipid contents of the two microalgae were increased. The maximum glycolipid content of *C. pyrenoidosa* and *Synechococcus* sp. at nitrate absence were as high as 24.61% and 15.37% (percentage of dry cell weight), which were 2.1 and 2.4 times than that in nitrate rich (11.81% and 6.50%) culture, respectively (Fig. 2a,c). While under different hydrophosphate levels, it is interesting to note that the maximum glycolipid content of *C. pyrenoidosa* (17.19%) was obtained in cells grown in hydrophosphate concentration of 0.01 g L^−1^, which was 1.5 times than that in hydrophosphate rich (11.29%) culture (Fig. 2c), suggesting that nitrogen and phosphorus have different influence on *C. pyrenoidosa* glycolipid accumulation. The maximum glycolipid content of *Synechococcus* sp. were found in cells grown in hydrophosphate concentration of 0 g L^−1^ (10.99%), which was 2.1 times than that in hydrophosphate rich (5.28%) culture (Fig. 2d). Previous study also showed that nitrate limitation induced an increase in glycolipid content to about 6.67% in *Haematococcus pluvialis*^13^. In microalgae *Chlorella* sp., the total glycolipid content increased to 10% by phosphate limitation^14^. These results suggested that *C. pyrenoidosa* and *Synechococcus* sp. have higher content of glycolipid.

The possible reason towards increase in glycolipid content in nitrogen and phosphorus limitation is that glycolipid makes up the decrease of other kinds of lipid in order to maintain normal metabolic function. Li *et al*.^19^ reported that nitrogen limitation could cause the decreasing of cellular content of thylakoid membrane activation of acyl hydrolase and stimulation of the phospholipid hydrolysis. Phosphorus deficiency can cause a drastic reduction in membrane phospholipids and replacement of these compounds by non-phosphorus glycolipids and sulfolipids^25^.

The concentrations of nitrogen and phophorus present in water are considered the fundamental factors and have direct influence on microalgal lipid accumulation^19^. Many researchers have already found that nitrogen and phophorus limitation or starvation will result an increase in microalgae lipid^12^,^26^,^27^. In this study, the similar phenomenon was also found in *C. pyrenoidosa* and *Synechococcus* sp. The effects of different nitrate and hydrophosphate concentrations on lipid content of the two microalgae were shown in Fig. 2. As Fig. 2 showed, the reduction of nitrate and hydrophosphate concentration in the medium could significantly increase the lipid content of the two microalgae. For *C. pyrenoidosa*, the lipid content was 52.03% in nitrate free culture, which was 1.5 times than that in the rich nitrate culture (34.68%) (Fig. 2a), while the lipid content was 49.80% in hydrophosphate free culture, which was 1.7 times than that in the rich hydrophosphate culture (29.56%) (Fig. 2b). For *Synechococcus* sp., under 0 g L^−1^ nitrate concentration and 0 g L^−1^ hydrophosphate concentration, the lipid content were 27.41% and 22.81%, which were 1.5 and 1.3 times than that in nitrate (18.90%) and hydrophosphate (17.06%) rich culture, respectively (Fig. 2c,d). Nitrogen limitation or starvation will result in decrease in photosynthesis and increase in lipid^28^. Phosphorus is also an essential component for microalgae metabolism, since its deficiency could affect the biochemical synthesis^29^. These results suggest that nitrogen and phosphorus deficiency are effective triggers to induce lipid accumulation in microalgae.

Although the glycolipid contents were high under nitrogen and phosphorus limitation or starvation conditions, the biomass concentration reduced with the reduction of nitrogen and phosphorus concentrations (Fig. 1, Table 1). In actual industrial production, both of glycolipid content and biomass concentration may be taken into overall consideration. Glycolipid productivity may have more reference value in actual production. Therefore, glycolipid productivities of the two microalgae were further investigated. As it showed in Table 1, the highest glycolipid productivity values of *C. pyrenoidosa* (18.20 mg L^−1^ d^−1^) and *Synechococcus* sp. (10.59 mg L^−1^ d^−1^) were obtained under nitrate concentration of 0.3 g L^−1^ and 0.8 g L^−1^, respectively. For different hydrophosphate levels, *C. pyrenoidosa* and *Synechococcus* sp. showed the highest values of glycolipid productivity of 13.29 mg L^−1^ d^−1^ and 7.97 mg L^−1^ d^−1^, under 0.01 g L^−1^ and 0.02 g L^−1^ hydrophosphate concentrations, respectively. Under these conditions, glycolipid productivities reached higher, meanwhile it could be attributed to little changes of biomass production.

### The analysis of glycolipid

Microalgae glycolipids are mainly consisted of MGDG, DGDG and SQDG. In order to further understand the effects of nitrogen and phosphorus limitation on their accumulation, the contents of MGDG, DGDG and SQDG were investigated under different nitrate and hydrophosphate levels. Table 2 showed the effects of different nitrate and hydrophosphate levels on MGDG, DGDG and SQDG contents for *C. pyrenoidosa* and *Synechococcus* sp. In general, the reduction of nitrate concentration ranging from 1.5 to 0 g L^−1^ induced an increase in MGDG, DGDG and SQDG contents of the two microalgae (Table 2). For *C. pyrenoidosa*, the MGDG, DGDG and SQDG contents increased to 44.00%, 18.04% and 29.02% at nitrate absence, which were 24.4%, 33.8% and 32.8% higher than that in nitrate rich culture (35.36%, 13.48% and 21.86%), respectively (Table 2). For *Synechococcus* sp., the MGDG, DGDG and SQDG content at nitrate absence were 50.2%, 29.88% and 18.93%, which were 8.5%, 23.2% and 12.1% higher than that in nitrate rich (46.28%, 24.25% and 16.89%) culture, respectively (Table 2). Nitrogen limitation improved the contents of three kinds of glycolipid, which may result in a significant increase of the total glycolipid (Fig. 2a,c). Under the deprivation of nitrogen, the photosynthetic capacity decreased, as well as the intracellular content of chlorophyll and chloroplast, which may lead the changes of photosynthetic membrane components. Previous studied showed that in *Chlorella* UTEX29^15^, nitrogen deficiency lead to a slight decrement in MGDG content and an increase in DGDG content to about 20%; whereas in *Oscillatoria splendida*^16^, nitrogen deficiency lead to a decrement in SQDG content. These results suggest that nitrogen limitation or starvation may affect the content of MGDG, DGDG and SQDG, but the influence is species dependent.

Phosphorus is also an important factor that can change the content of membrane lipid. Geiger^30^ mentioned that phosphate deprivation resulted in the accumulation of different phosphate free lipids. During phosphate deprivation, the amount of phosphate that combines to phospholipids in membranes is reduced to sustain other cellular processes (e.g. DNA and RNA synthesis)^31^. As Table 2 showed, the reduction of hydrophosphate concentration ranging from 0.04 to 0 g L^−1^ induced an increase in DGDG and SQDG content, while a decrement in MGDG content of the two microalgae. The similar regulation was also reported in plant: under phosphate-limiting conditions, the amounts of DGDG and SQDG increase, whereas the amount of MGDG remains constant of slightly decreases^31^. For *C. pyrenoidosa*, the highest DGDG and SQDG content obtained at hydrophosphate absence were 21.17% and 34.96%, which were 69.2% and 48.4% higher than that in hydrophosphate rich culture (12.51% and 23.56%), respectively (Table 2). However, the MGDG content showed 24.5% decrease as hydrophosphate concentration reduced from 0.04 g L^−1^ (37.60%) to 0 g L^−1^ (28.39%) (Table 2). This was different from the influence on MGDG, DGDG and SQDG contents made by different nitrate levels, which may be a reason to explain the different influence of nitrate and hydrophosphate on glycolipid contents for *C. pyrenoidosa* (Fig. 2a,c). *Synechococcus* sp. showed the similar regulation, the highest DGDG and SQDG content obtained at hydrophosphate absence were 32.14% and 26.34%, which were 21.2% and 71.3% higher than that in hydrophosphate rich culture (26.52% and 15.38%), respectively; whereas the MGDG content have 22.2% decrease from 44.87% (0.04 g L^−1^ hydrophosphate) to 34.92% (0 g L^−1^ hydrophosphate) (Table 2). Similar phenomenon could also be found in microalgae *Synechocystis* sp. PCC 6803^17^, where the contents of MGDG, DGDG and SQDG were 49.7%, 25.9% and 14.3% in phosphorus rich culture, 38.8%, 30.2% and 20.2% in phosphorus starvation culture, respectively. Benning *et al*.^32^ and Ohta *et al*.^2^ also mentioned that phosphate concentration in growth medium strongly influences DGDG and SQDG content in cyanobacteria. These results suggest that microalgae should be similar to higher plants that increase the content of DGDG and SQDG when grown on phosphorus limitation or starvation conditions.

It is interesting to note that MGDG, DGDG and SQDG content changed slightly with the reduction of nitrate concentration ranging from 1.5 to 0.5 or 0.3 g L^−1^, suggesting that the constitution of main glycolipid in the two microalgae changed less under such conditions. However, the three glycolipid contents sharply increased in lower nitrate concentrations (0.3 or 0 g L^−1^) (Table 2). The similar regulation could also be found in DGDG and SQDG content under different hydrophosphate concentrations. As shown in Table 2, DGDG and SQDG content of the two microalgae were either maintained or increased slightly under 0.02–0.04 g L^−1^ hydrophosphate, but changed sharply when hydrophosphate concentration decreased to 0.01 or 0 g L^−1^. These results suggested that microalgae could maintain glycolipid constitution within certain limitation of nitrogen and phosphorus concentrations, and lower nitrogen (phosphorus) concentrations or nitrogen (phosphorus) starvation were more likely to induce a sharp change in MGDG, DGDG and SQDG contents of microalgae.

### Centre-complex test to optimize glycolipid production condition

**Figure 3:**
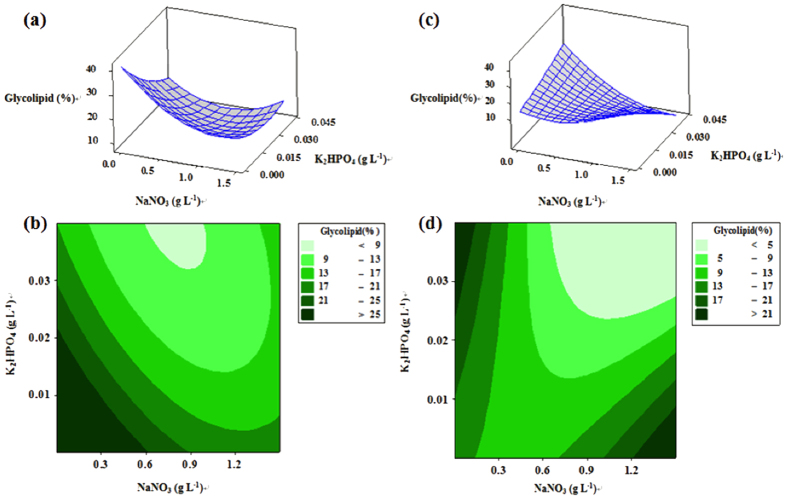
Response surface and contour plots. (**a**) Response surface of different nitrate concentrations and hydrophosphate concentrations on glycolipids contents of *Chlorella pyrenoidosa*. (**b**) Contour plots of different nitrate concentrations and hydrophosphate concentrations on glycolipids contents of *Chlorella pyrenoidosa*. (**c**) Response surface of different nitrate concentrations and hydrophosphate concentrations on glycolipids contents of *Synechococcus* sp. (**d**) Contour plots of different nitrate concentrations and hydrophosphate concentrations on glycolipids contents of *Synechococcus* sp.

As it mentioned above, glycolipid content of the two microalgae was investigated under different nitrate and hydrophosphate levels, respectively. However, the simultaneous action of nitrate and hydrophosphate limitation on microalgae glycolipid accumulation was still unknown. Thus, centre-complex test was used to find out that under which nitrate and hydrophosphate condition the glycolipid accumulation could be affected most. Table 3 showed the experimental values of glycolipid content and calculated values of glycolipid content in centre-complex test of *C. pyrenoidosa* and *Synechococcus* sp. According to it, the experimental values did not appear to be much difference to the calculated values. These results suggested that centre-complex test on glycolipid of the two microalgae be creditable. Figure 3 showed the response surface and contour plots of different nitrate and hydrophosphate concentrations on glycolipids of *C. pyrenoidosa* and *Synechococcus* sp., respectively. As it showed, low nitrate hydrophosphate levels (0–0.6 g L^−1^ nitrate concentration and 0–0.021 g L^−1^ hydrophosphate concentration) were suitable for *C. pyrenoidosa* glycolipid accumulation (Fig. 3a,b). Under this condition, the glycolipid content could reach more than 25%; while under nitrate or hydrophosphate limitation condition, the glycolipid content was 18–24%. These results indicated that the glycolipid content was further increased under both nitrate and hydrophosphate limitation conditions for *C. pyrenoidosa*. The similar phenomenon was also found in *Synechococcus* sp. More than 21% glycolipid content was obtained under the conditions of lower nitrate (0–0.15 g L^−1^) and higher hydrophosphate (0.025–0.04 g L^−1^) or higher nitrate (1.21–1.5 g L^−1^) and lower hydrophosphate (0–0.007 g L^−1^) levels (Fig. 3c,d), while under nitrate or hydrophosphate limitation condition, the glycolipid content was only 10–15%. These results suggest that simultaneous nitrate and hydrophosphate limitation in culture medium could make further improvement of glycolipid accumulation in microalgae.

## Conclusions

The present study showed nitrogen and phosphorus limitation was an effective way for stimulating glycolipid accumulation and altering MGDG, DGDG, SQDG contents. Maximum glycolipid productivities were obtained at higher nitrate (≥0.3 g L^−1^) or hydrophosphate (≥0.01 g L^−1^) concentrations. MGDG, DGDG, SQDG contents improved slightly under nitrate starvation, whereas hydrophosphate deprivation resulted in significant increases in contents of DGDG and SQDG, decrease in MGDG. *C. pyrenoidosa* cultivated with lower nitrate and hydrophosphate concentrations had higher glycolipid content (>25%). *Synechococcus* sp. cultivated with lower nitrate and higher hydrophosphate concentration or higher nitrate and lower hydrophosphate concentration obtained higher glycolipid content (>21%).

## Methods

### Microalgae cultures

The microalgae species *Chlorella pyrenoidosa* and *Synechococcus* sp. were cultivated in BG-11 medium containing (g L^−1^) NaNO_3_, 1.5; K_2_HPO_4_, 0.04; MgSO_4_·2H_2_O, 0.075; CaCl_2_·2H_2_O, 0.036; citric acid, 0.006; ferric ammonium citrate, 0.006; EDTA, 0.001; Na_2_CO_3_, 0.020 and 1 mL of micronutrient solution containing (g L^−1^) H_3_BO_3_, 2.86; MnCl_2_·4H_2_O, 1.81; ZnSO_4_·7H_2_O, 0.222; NaMoO_4_·5H_2_O, 0.390; CuSO_4_·5H_2_O, 0.0790; Co(NO_3_)_2_·6H_2_O, 0.0494.

In the experiments, *C. pyrenoidosa* and *Synechococcus* sp. were cultivated in 1 L Erlenmeyer flask (20 cm length, 10 cm diameter) with 400 mL working volume of modified BG-11 medium under 25 ± 2 °C and 140 μmol/m^2^/s. The light intensity was measured by a light meter (TES-1399, TES Electrical Electronic Corp., China). The initial pH was 8.0. For the experiment about nitrate condition, cells were grown with different initial nitrate concentrations of 0, 0.3, 0.5, 0.8, 1.5 g L^−1^. For the experiment about hydrophosphate condition, cells were grown with different initial hydrophosphate concentrations of 0, 0.01, 0.02, 0.03, 0.04 g L^−1^.

### Determination of growth and biomass production

The dry cell weight (g L^−1^) was measured according to the method as described by Chiu *et al*.^33^. *C. pyrenoidosa* and *Synechococcus* sp. were harvested by centrifugation (5804R, Eppendorf, Germany) at 8000 rpm and 11000 rpm for 10 min, respectively, and washed twice with distilled water. The pellet was lyophilized drying in a freeze drier (FD-1–50, Boyikang, China) for dry weight measurement. A calibration curve of OD_680_ for *C. pyrenoidosa* and OD_730_ for *Synechococcus* sp. versus cell density was constructed from samples that ranged from OD_680_ 0.3–4.0 and OD_730_ 0.5–6.0, respectively. Each sample was diluted to give an absorbance in the range of 0.1–1.0 if the optical density was greater than 1.0. Cell density of *C. pyrenoidosa* was calculated using the equation: Cell density (g L^−1^) = 0.308 × OD_680_ − 0.046 (R^2^ = 0.996). For *Synechococcus* sp., the equation was Cell density (g L^−1^) = 0.279 × OD_730_ + 0.066 (R^2^ = 0.990). Therefore, the optical density can be used to precisely predict the biomass concentration. The dry cell weights were the means of three independent experiments, the error bars were plus minus standard deviation.

### Lipid extraction and quantification

The total lipids were extracted from microalgae cells using a modified method of Zhu *et al*.^34^. Freeze-dried algae power (0.2 g) was suspended in 10 mL solvent mixture of chloroform: methanol (2:1, v/v). After stirring for 10 min, the samples were centrifuged at 8000 rpm for 10 min. The procedure was repeated three times until the total lipids were fully extracted. The solvent phase was transferred and evaporated in a water bath at 5°C. Then the total lipids were weighed using analytical balance (BS 124S, Sartorius, Germany). Lipid contents were the means of three independent experiments, the error bars were plus minus standard deviation.

### Glycolipid extraction and quantification

The total glycolipids were extracted from microalgae cells using a method from Damiani *et al*.^13^. 5 mL chloroform was used to dissolve the extracted lipid. Then add it to a silicagel column. 25 mL solvent mixture of acetone: methanol (9:1, v/v) was added to the column to elute, then gather the glycolipids. The solvent phase was transferred and evaporated in a water bath at 55 °C. Then the total glycolipids were weighed using analytical balance (BS 124S, Sartorius, Germany). Glycolipid contents were the means of three independent experiments, the error bars were plus minus standard deviation. The glycolipid productivity *P* (mg L^−1^ d^−1^) was calculated according to the following Eq. (1):


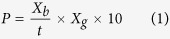


where *X*_*b*_ is the dry cell weight concentration (g L^−1^) and *X*_*g*_ is the glycolipid content (%) at time *t* (d).

### The glycolipid analysis

The method of Xue *et al*.^35^ was used to analyze the glycolipid. MGDG and DGDG were isolated by silica gel column with the elution solvent, chloroform: methanol: acetone: acetic acid (73:1.5:25:0.5, v/v/v/v) and acetone: benzene: water (91:30:4, v/v/v) respectively. The solvent phase was transferred and evaporated in a water bath at 55 °C, then weighed using analytical balance (BS 124S, Sartorius, Germany). SQDG was isolated on a silica gel column with the elution system chloroform: methanol: methanoic acid (100:18:3.5, v/v/v), and the fractions containing SQDG were obtained. The combined mixture was then applied to second column and washed with chloroform: methanol (95:5, v/v) and chloroform: methanol (90:10, v/v). SQDG were obtained by elution with chloroform: methanol (80:20, v/v). The solvent phase was transferred and evaporated in a water bath at 55°C. Then SQDG was weighed using analytical balance (BS 124S, Sartorius, Germany). MGDG, DGDG and SQDG contents were the means of three independent experiments, the error bars were plus minus standard deviation.

### Design of centre-complex test

The software Minitab 15 was used to design the centre-complex test about the two factors of nitrate and hydrophosphate levels. Coded factors and levels of response surface design were shown in Table 4. The response surface for *C. pyrenoidosa* was calculated using the equation: Glycolipid content (%) = 41.38 – 35.09 × N – 936.47 × P + 13.61 × N^2^ + 8095.39 × P^2^ + 341.75 × N × P; For *Synechococcus* sp., the equation was: Glycolipid content (%) = 14.43 – 12.46 × N + 227.11 × P + 14.77 × N^2^ + 2045.79 × P^2^ – 772.47 × N × P, where N is the NaNO_3_ concentration (g L^−1^) and P is the K_2_HPO_4_ concentration (g L^−1^).

## Additional Information

**How to cite this article**: Wang, X. *et al*. Nitrogen and hydrophosphate affects glycolipids composition in microalgae. *Sci. Rep.* **6**, 30145; doi: 10.1038/srep30145 (2016).

## Figures and Tables

**Table 1:** The maximum biomass concentration (*Xmax*) and glycolipid productivity (*P*) of *Chlorella pyrenoidosa* (Cp) *and Synechococcus* sp. (Ss) under different nitrate and hydrophosphate concentrations (means ± SD).

NaNO_3_ Concentration (g L^−1^)	*Xmax*(g L^−1^)		*P*(mg L^−1^ d^−1^)		K_2_HPO_4_ Concentration (g L^−1^)	*Xmax*(g L^−1^)		*P*(mg L^−1^ d^−1^)	
	Cp	Ss	Cp	Ss		Cp	Ss	Cp	Ss
1.5	0.97 ± 0.01	1.81 ± 0.01	8.19 ± 0.15	7.36 ± 0.52	0.04	1.08 ± 0.04	1.83 ± 0.03	8.65 ± 0.37	6.04 ± 0.90
0.8	0.91 ± 0.03	1.76 ± 0.01	7.11 ± 1.02	10.59 ± 2.04	0.03	1.09 ± 0.05	1.66 ± 0.08	7.32 ± 0.67	7.54 ± 0.32
0.5	1.02 ± 0.01	1.33 ± 0.01	8.95 ± 1.59	8.93 ± 0.74	0.02	1.44 ± 0.03	1.30 ± 0.05	10.73 ± 0.38	7.97 ± 0.82
0.3	1.30 ± 0.04	1.02 ± 0.03	18.20 ± 2.52	8.45 ± 1.21	0.01	1.08 ± 0.01	0.89 ± 0.05	13.29 ± 1.49	4.43 ± 0.55
0	0.74 ± 0.01	0.28 ± 0.01	13.00 ± 2.30	2.72 ± 0.21	0	0.31 ± 0.01	0.29 ± 0.01	3.21 ± 0.27	1.97 ± 0.25

**Table 2:** The MGDG, DGDG and SQDG contents (percentage of total glycolipid) of *Chlorella pyrenoidosa and Synechococcus* sp. under different nitrate and hydrophosphate concentrations (means ± SD).

	Chlorella pyrenoidosa			Synechococcus sp.		
	MGDG (%)	DGDG (%)	SQDG (%)	MGDG (%)	DGDG (%)	SQDG (%)
NaNO_3_ Concentration (g L^−1^)						
1.5	35.36 ± 0.96	13.48 ± 1.36	21.86 ± 1.67	46.28 ± 2.60	24.25 ± 1.21	16.89 ± 0.40
0.8	36.47 ± 0.52	13.65 ± 1.35	22.56 ± 2.35	44.90 ± 1.12	23.76 ± 1.63	16.03 ± 1.43
0.5	37.92 ± 1.39	14.77 ± 1.37	23.03 ± 2.08	43.33 ± 0.96	25.37 ± 1.27	17.61 ± 2.18
0.3	38.02 ± 1.49	16.89 ± 0.67	27.14 ± 0.35	45.64 ± 0.72	25.26 ± 2.37	17.33 ± 2.35
0	44.00 ± 1.20	18.04 ± 1.19	29.02 ± 1.79	50.20 ± 1.50	29.88 ± 1.27	18.93 ± 0.43
K_2_HPO_4_ Concentration (g L^−1^)						
0.04	37.60 ± 1.00	12.51 ± 1.41	23.56 ± 1.47	44.87 ± 1.53	26.52 ± 1.00	15.38 ± 1.03
0.03	30.27 ± 1.90	12.62 ± 2.03	24.04 ± 0.57	43.48 ± 1.27	26.33 ± 1.54	15.00 ± 0.93
0.02	29.01 ± 0.73	15.73 ± 0.81	25.01 ± 0.87	40.75 ± 1.04	26.94 ± 1.39	17.75 ± 1.21
0.01	27.50 ± 1.80	17.63 ± 0.83	29.64 ± 0.92	34.75 ± 6.16	30.16 ± 0.95	23.54 ± 0.92
0	28.39 ± 2.01	21.17 ± 1.98	34.96 ± 1.34	34.92 ± 1.83	32.14 ± 1.75	26.34 ± 1.18

**Table 3:** The experimental values of glycolipid content (GE) in experiment and calculated value of glycolipid content (GC) in centre-complex test of *Chlorella pyrenoidosa and Synechococcus* sp.

NaNO_3_(g L^−1^)	K_2_HPO_4_(g L^−1^)	*Chlorella pyrenoidosa*		*Synechococcus* sp.	
		GE (%)	GC (%)	GE (%)	GC (%)
0.3	0.01	22.05	24.55	8.34	11.88
0.8	0.01	17.13	16.20	13.35	9.91
0.3	0.03	12.65	14.35	9.21	13.42
0.8	0.03	10.88	9.41	6.72	3.73
0	0.02	27.9	25.89	24.09	19.49
1.5	0.02	13.57	14.13	9.89	10.86
0.5	0	27.79	27.24	11.92	11.59
0.5	0.04	9.45	9.57	9.39	8.50
0.5	0.02	17.47	15.16	9.66	9.23
0.5	0.02	16.68	15.16	8.14	9.23
0.5	0.02	14.75	15.16	10.49	9.23
0.5	0.02	12.67	15.16	8.46	9.23
0.5	0.02	14.1	15.16	9.71	9.23

**Table 4:** Coded factors and levels of response surface design.

Factors	Independent variable	Coded levels				
		−1.414	−1	0	1	1.414
NaNO_3_ (g L^−1^)	*X*_*2*_	0	0.3	0.5	0.8	1.5
K_2_HPO_4_ (g L^−1^)	*X*_*3*_	0	0.01	0.02	0.03	0.04

Where *X*_*2*_ and *X*_*3*_ are the independent variable of NaNO_3_ and K_2_HPO_4_ concentrations, respectively.
